# Kentucky Correspondent

**Published:** 1871-04

**Authors:** 


### KENTUCKY CORRESPONDENT.

"*Drs. Powell & Goldsmith.—Gentlemen*:—Let me congratulate you on the handsome appearance and quality of your Companion. It certainly deserves success, for it will certainly do good. No doctor can read it without being profited."

If the reader knew the source of the above extract, he would not be surprised should we tell him how much gratification it afforded us to receive it. It comes from a large-souled, high-toned brother of Kentucky, eminent in all the grand characteristics of massive intellect and professional distinction. A man, like King Saul, who stands a full "head and shoulders" above thousands of able men of either hemisphere.

Our brother occupies a proud position—one he fills with honor. As a writer, his power and influence is acknowledged and felt. He is a valient gladiator—a foreman worthy of any man's steel. He wields a glittering sword, with a sturdy arm. Will he not strike high? Will he not battle against "principalities and powers"; against "spiritual wickedness in high places," in the professional arena? We feel confident he will. Kentucky expects it; Georgia would thank him, and the profession honor him, for it.

We shall not be able to accompany him to California to greet our brethren in that great brotherhood of the profession, the National Association; but our heart will go with him. We bid him, and all, God-speed in their efforts to uphold the honor and promote the interests of the profession. May a new era dawn upon our cause as the results of their deliberations.

